# Neutrophil to lymphocyte ratio and principal component analysis offer prognostic advantage for dogs with mammary tumors

**DOI:** 10.3389/fvets.2023.1187271

**Published:** 2023-06-16

**Authors:** Eileen Uribe-Querol, Laura Romero-Romero, Tzipe Govezensky, Carlos Rosales

**Affiliations:** ^1^Laboratorio de Biología del Desarrollo, División de Estudios de Posgrado e Investigación, Facultad de Odontología, Universidad Nacional Autónoma de México, Mexico City, Mexico; ^2^Departamento de Patología, Facultad de Medicina Veterinaria y Zootecnía, Universidad Nacional Autónoma de México, Mexico City, Mexico; ^3^Apoyo de estadística, Instituto de Investigaciones Biomédicas, Universidad Nacional Autónoma de México, Mexico City, Mexico; ^4^Departamento de Inmunología, Instituto de Investigaciones Biomédicas, Universidad Nacional Autónoma de México, Mexico City, Mexico

**Keywords:** neutrophil, lymphocyte, breast cancer, canine mammary tumor, inflammation, cancer, albumin, globulin

## Abstract

**Introduction:**

In veterinary medicine, cancer is the leading cause of death in companion animals, and mammary gland tumors represent the most common neoplasm in female dogs. Several epidemiological risk factors, such as age, breed, hormones, diet, and obesity have been reported to be relevant for canine mammary tumors. Nowadays, the gold standard for diagnosis of canine mammary tumors is the pathological examination of the suspected tissue. However, tumor grade can only be assessed after surgical removal or biopsy of the altered tissue. Therefore, in cases of tumors that could be surgically removed, it would be very helpful to be able to predict the biological behavior of the tumor, before performing any surgery. Since, inflammation constitutes part of the tumor microenvironment and it influences each step of tumorigenesis, cellular and biochemical blood markers of systemic inflammation, such as the neutrophil to lymphocyte ratio (NLR) and the albumin to globulin ratio (AGR) have been proposed as prognostic factors for human cancer development. The NLR and the AGR have not been explored enough as prognostic factors for cancer development in veterinary medicine.

**Methods:**

To determine the prognostic value of NLR in canine mammary tumors, clinical records including biochemistry and hematological studies of female dogs with mammary tumors and of control healthy dogs, were used to determine the pre-treatment NLR and AGR. Other clinical data included age, breed, tumor size, histological tumor grade, and survival time after surgery.

**Results and discussion:**

It was found that a higher pre-treatment NLR value (NLR > 5) associates with less survival rate. In contrast, the AGR did not show any predictive value on the malignancy of the tumor. However, by combining the NLR with AGR, age of the dog, and tumor size in a principal component analysis (PCA), the grade of the tumor and survival after surgery could be appropriately predicted. These data strongly suggest that pre-treatment NLR values have a prognostic value for the survival rate after surgery of dogs with mammary tumors.

## 1. Introduction

In veterinary medicine, dogs are nowadays more than just companion animals. They have become family members and therefore more attention is invested in their welfare. Cancer is the leading cause of death in companion animals, and mammary gland tumors represent the most common neoplasm in female dogs (1). Dogs develop mammary gland tumors and other cancer types spontaneously, similarly to humans, in the presence of an intact immune system (2). These mammary tumors also exhibit many clinical and molecular similarities to human breast cancer (1, 3). Both dogs and humans share environmental risk factors for cancer (4), and in both species, cancer-induced mortality persists as a crucial problem, particularly in cases with late diagnosis (2, 5). In dogs, mammary cancer is multifactorial with various aspects contributing to its initiation and development (3). Several epidemiological risk factors, such as age, breed, hormones, diet, and obesity have been reported to be relevant for canine mammary tumors (1, 3). The annual incidence of canine mammary tumors varies considerably among different studies. In Mexico, it was reported to be 17% with half of the tumors being malignant (6), while in Italy up to 54% of all tumors in female dogs were mammary tumors (7). More recently, the incidence in China was reported to be 46.7% (8), while in New Zealand it was 56% (9), and in Germany it was 64.7% (10). Thus, generally the global incidence for canine mammary tumors is 50%. These tumors appear more frequently in older dogs and in pure breeds (6, 7, 11).

At present, the gold standard for diagnosis of canine mammary tumors is the pathological examination of the suspected tissue. Histological examination is also required for establishing the histopathological grade of malignancy (tumor grade) and the proliferation (mitotic) index, and by this means estimate the prognosis (12). However, tumor grade and mitotic index can only be assessed after surgical removal or biopsy of the altered tissue (13). Therefore, in cases of tumors that could be surgically removed, it would be very helpful to be able to predict the biological behavior of the tumor, before performing any surgery, particularly when dog owners' finances are limited. A biomarker that suggests that an abnormal growth is more likely to be either benign or malignant would provide an early instrument for better treatment planning (14). A number of pretreatment markers, including age, race, tumor size, and ulceration, have been taken into consideration for their potential value in predicting the biological behavior of the tumor and for making therapeutic decisions (7, 11, 15, 16). These markers are susceptible to varying degrees of subjectivity and hence have led to inconsistent results.

Inflammation is now recognized as a hallmark for cancer development (17, 18). Since, inflammation constitutes part of the tumor microenvironment, it influences each step of tumorigenesis, including tumor initiation, growth, and metastatic progression (19, 20). In addition, the presence of a systemic inflammatory response is associated with reduced survival of cancer patients. Therefore, cellular and biochemical blood markers of systemic inflammation, such as the neutrophil to lymphocyte ratio (NLR) (that is, the ratio between the total blood neutrophil count and the total blood lymphocyte count) and the albumin to globulin ratio (AGR) (that is, the ratio between albumin concentration and globulins concentration in blood), have recently emerged as prognostic factors for human cancer development (21, 22). The NLR and the AGR have not been explored enough as prognostic factors for cancer development in veterinary medicine (23). Although, the NLR was reported not to be a useful prognostic biomarker for canine lymphoma (24) or oral melanoma (25), it was found to be able to differentiate between soft-tissue sarcomas and benign mesenchymal neoplasia (26), as well as between high- and low-grade canine mast cell tumors (13, 27). Also, the NLR correlated with survival time in dogs with diffuse large B-cell lymphoma (28), and in dogs with lower urinary tract urothelial carcinoma (29). Moreover, the NLR has also been suggested as an independent prognostic marker for feline mammary carcinomas (30, 31), and for feline injection-site sarcoma (32). In the case of canine mammary tumors there are not studies about the potential prognostic value of NLR. Thus, to determine the prognostic value of NLR in canine mammary tumors, clinical records including biochemistry and hematological studies of female dogs with mammary tumors that underwent surgery and of control healthy dogs, were used to determine the pre-treatment NLR and AGR. Other clinical data included age, breed, tumor size, histological tumor grade, and survival time after surgery. Our data strongly suggest that pre-treatment NLR values have a prognostic value for the survival rate after surgery of dogs with mammary tumors.

## 2. Materials and methods

### 2.1. Study subjects

The participants of this retrospective cohort study were selected from 105 female dogs with mammary tumors that underwent lumpectomy or mastectomy with the consent of their owner, at the Teaching Animal Hospital of the Veterinary School (Facultad de Medicina Veterinaria y Zootecnia) at the University of Mexico (Universidad Nacional Autónoma de México - UNAM) from February 2012 to October 2013. Forty-five female dog patients with mammary tumors fulfilled the inclusion criteria of having clinical records that included hematology and biochemistry studies within 2 weeks prior to surgery and a complete histological study to grade the tumors (Table 1) and were entered in this study. Also, twenty-five healthy dogs (Table 2) that visited the clinic for routine check-ups or to receive vaccinations, and that had clinical records that included hematology and biochemistry studies were entered in this study. Included control animals were younger than tumor-bearing animals, because many older dogs although did not have tumors, usually presented other alterations that varied their cellular and biochemical parameters and thus they did not fulfill the inclusion criteria. Clinical staging of dogs was based on tumor size, according to the World Health Organization staging scheme for dogs (33), as follows stage I, animals with tumor smaller than 2 cm; stage II, animals with tumor between 2 and 4 cm; stage III, animals with tumor larger than 4 cm. All animals with tumors, independently of their stage, went into surgery to remove the tumor. Clinical data also comprised breed, age, tumor size (maximum length), and tumor grade (Table 1). Dogs were pure breeds or crossbreeds and ranged from 3 to 15 years of age (mean age: 10.5 years). Patients with a concurrent illness or that had received medication (such as corticosteroids or phenobarbital) that could alter biochemical or cellular parameters were excluded from participation. Approval by the Committee for the Care of Experimental Animals was not required, since this is a retrospective study that did not include experimental animals, and all dogs underwent laboratory tests and surgery for medical treatment with the approval from their owners.

**Table 1:** Characteristics of female dogs and their mammary tumors.

**Breed**	**Histological type of tumor**	**Tumor grade**	**Tumor size (cm)**	**Age (years)**	**NLR**	**Survival (months)**
Dachshund	Benign mixed tumor	Benign	0.8	7	2.13	18
Miniature Pinscher	Benign mixed tumor	Benign	1.5	9	2.44	18
Schnauzer	Benign mixed tumor	Benign	0.8	9	4.5	18
Cocker	Complex adenoma	Benign	1.8	9	3.1	18
Chihuahua	Lobular hyperplasia	Benign	0.5	8	3.75	18
Dachshund	Simple adenoma	Benign	6	13	3.38	18
Great dane	Carcinoma *in situ*	G I	0.5	3	8.3	18
Poodle	Tubulopapillary carcinoma	G I	3	9	3.1	18
Poodle	Tubulopapillary carcinoma	G I	1.4	4	1.6	18
Rottweiler	Carcinoma-mixed type	G I	1	4	7.4	18
Poodle	Tubular carcinoma	G I	6	12	2.75	18
Schnauzer	Tubulopapillary carcinoma in simple adenoma	G I	0.8	9	2.09	18
Poodle	Tubulopapillary carcinoma in cystic adenoma	G I	1.2	9	3.05	18
Rottweiler	Papillary carcinoma	G I	0.5	6	5.21	18
Labrador	Carcinoma-mixed type in benign mixed tumor	G II	15	12	4.58	18
Cocker	Carcinoma-mixed type	G II	7	13	31	18
Poodle	Mucinous carcinoma	G II	2	11	2.11	18
Labrador	Carcinoma-mixed type	G II	4	8	2.18	18
Labrador	Carcinoma-mixed type	G II	3	9	19	18
Mixed-breed	Papillary carcinoma	G II	5	12	4.0	8
Poodle	Carcinoma-mixed type	G II	5	13	16.4	18
Poodle	Carcinoma-mixed type	G II	3	8	4.0	18
Samoyed	Cribriform carcinoma	G II	3	15	13.5	3
Dachshund	Carcinoma-complex type	G II	3	12	2.38	18
Poodle	Micropapillary carcinoma	G II	3	13	4.65	18
Poodle	Carcinoma-complex type	G II	2	9	32.2	18
Husky	Tubulopapillary carcinoma	G II	2	8	4.29	18
Mixed-breed	Carcinoma-mixed type	G III	3	11	7.36	18
Whelsh corgi	Solid carcinoma	G III	7	15	5.24	6
Labrador	Osteosarcoma	G III	18	8	6.61	1
Cocker	Carcinoma-mixed type	G III	18	15	11.1	2
Labrador	Carcinosarcoma	G III	7	11	2.69	18
Cocker	Carcinosarcoma	G III	4	14	8.21	18
Cocker	Simple carcinoma	G III	16	12	2.37	3
Labrador	Carcinosarcoma	G III	5	10	6.0	18
Poodle	Carcinoma-mixed type	G III	4	15	6.53	8
Mixed-breed	Solid carcinoma	G III	4	12	4.09	18
Mixed-breed	Carcinoma-mixed type	G III	0.5	13	4.29	18
Poodle	Solid carcinoma	G III	0.5	12	4.47	18
Poodle	Carcinosarcoma, Carcinoma-complex type	G III	2	10	2.47	18
Schnauzer	Solid carcinoma	G III	3	15	4.0	1
Cocker	Carcinosarcoma	G III	3.5	11	7.3	18
Husky	Tubulopapillary carcinoma	G III	2.5	8	18.5	13
Dachshund	Carcinoma-mixed type	G III	13	14	9.24	2
Poodle	Carcinosarcoma	G III	4	12	6.22	2

NLR, neutrophil to lymphocyte ratio.

**Table 2:** Characteristics of healthy female dogs.

**Breed**	**Age (years)**	**NLR**
Australian pastor	4	4.0
Beagle	2	1.5
Boxer	0.4	2.0
Chihuahua	3	4.27
Chihuahua	4	3.29
Chihuahua	8	6.57
Chihuahua	5	3.6
English setter	2	1.75
English setter	6	2.4
English shepherd	5	3.57
Great dane	7	12.1
Husky	4	3.1
Husky	4	9.5
Labrador	7	2.27
Mixed-breed	1	3.79
Mixed-breed	5	3.5
Mixed-breed	0.5	4.75
Mixed-breed	4	1.52
Pomerania	0.5	3.1
Pomerania	2	7.0
Poodle	9	1.92
Pug	2	3.1
Rottweiler	7	3.0
Schnauzer	6	3.35
Sharpei	4	1.4

NLR, neutrophil to lymphocyte ratio.

### 2.2. Histology

After surgical procedures, fresh tumor size (largest dimension) was determined. Tumor tissues were fixed for 48 h in 10 % buffered formalin. Next, tumors were trimmed, and embedded in paraffin. Histological sections of 5 μm were cut and processed for staining with hematoxylin and eosin (H&E). Sections were reviewed and tumors were graded according to Goldschmidt's criteria (12). In cases of a patient having several mammary tumors, the dog was classified in the highest-grade group, and the tumor included for further analysis was also the tumor with the highest grade (Table 1).

### 2.3. Laboratory data

Hematological parameters were obtained from blood samples collected prior to surgery. Blood was obtained in tubes containing ethylenediaminetetraacetic acid (EDTA) as anticoagulant and was processed in the Exigo^®^ veterinary hematological analyzer model Vet 52054 from Boule Diagnostics AB (Spånga, Sweden), using the impedance technique for determining the total leukocyte count. The leukocyte differential count was determined by microscopic examination (at 100X magnification) of blood smears stained with Wright solution. A minimum of two hundred leukocytes were counted in each sample to obtain the relative frequency of different leukocytes. The frequency of each leukocyte type and the total leukocyte count were used to calculate the total number of lymphocytes and neutrophils in international units (cells × 10^9/^L) (34) (Table 3). The neutrophil to lymphocyte ratio (NLR) was calculated by dividing the total neutrophil count by the total lymphocyte count.

**Table 3:** Reference value intervals for canine hematological parameters.

**Hematological parameter**	**Reference interval**
White blood cells	6.0–17.0 × 10^9^/L
Neutrophil count	3.0–11.5 × 10^9^/L
Lymphocyte count	1.0–4.8 × 10^9^/L
Albumin	29–40 g/L
Globulin	23–39 g/L

g, gram; L, liter.

Biochemical parameters from blood included in this study were the amount of total serum proteins: albumin and globulins. Determination of these proteins was carried out with the Dirui^®^ automatic analyzer Model CST-240 (Dirui Industrial Co., Ltd.; Changchun, Jilin, China) using colorimetric methods. The Biuret reaction was used for determining total proteins concentration, and the bromocresol green reaction for determining albumin concentration. Amount of globulins was estimated by subtracting albumin concentration from total proteins concentration. All protein concentrations were reported in international units (g/L) (Table 3). The albumin to globulin ratio (AGR) was then calculated by dividing the albumin concentration by the globulin concentration.

### 2.4. Survival time

Survival time was defined as the time (months) between surgical tumor removal and the time of death (end point) or the time of the last follow-up (18 months). Death provoked by tumor-associated causes and confirmed at post-mortem was recorded as data. Animals were excluded from analysis if their causes of death were unknown or were not related to the neoplastic process.

### 2.5. Statistical analysis

Quantitative data were expressed as mean ± standard error of mean (SEM). The survival curves were calculated using the Kaplan–Meier method. Empirical distribution was used to illustrate the distribution of the variables measured (such as NLR and AGR), and it was calculated with MATLAB, version R2017b from MathWorks, Inc. (Natick, MA, USA). Statistical analysis was performed with the SAS software (SAS Institute; Cary, NC, USA) using one-way ANOVA followed when appropriate, by a contrast test to compare none, benign, and grade 1 tumors vs. grade 2 and grade 3 tumors. Normality of data was analyzed using the Fisher-Pearson standardized third moment coefficient (35); a logarithmic transformation was needed to approach normality. Homogeneity of variance was analyzed using Levene's test. Receiver operating characteristic (ROC) curve analysis (36, 37) was performed with the SAS software (SAS Institute; Cary, NC, USA) to determine the cutoff value. Principal component analysis (PCA) (38, 39) was performed with the SAS software (SAS Institute; Cary, NC, USA). For PCA analysis, variables were standardized by subtracting their mean value and dividing by their standard deviation. Differences were considered statistically significant at a value *p* < 0.05.

## 3. Results

### 3.1. Dog characteristics of tumor-bearing female dogs

Forty-five female dogs with mammary gland tumors (Table 1) and twenty-five healthy female dogs (Table 2) were included in this study. Dog races varied from small dogs (e.g., Chihuahua) to big dogs (e.g., Husky). Tumor-bearing dogs presented variable number of mammary tumors, from one to three tumors each. Healthy dogs were much younger than sick dogs. This was a consequence of the inclusion criteria that required healthy animals with unaltered hematology and biochemistry studies. The average age of healthy dogs was 4.1 ± 0.5 years (Mean ± standard error; n = 25) (Table 2), while the average age of tumor-bearing dogs was 10.5 ± 0.4 years (Mean ± standard error; *n* = 45) (Table 1). Tumor malignancy was estimated by histological analysis and tumors were then classified according to Goldschmidt's criteria into benign, grade I (G I), grade II (G II), or grade III (G III). Dogs with benign tumors (n = 6) had tumors with an average size of 1.9 ± 0.8 cm, and an average age of 9.2 ± 0.8 years. Dogs with G I tumors (*n* = 8) had tumors with an average size of 1.8 ± 0.7 cm, and an average age of 7.0 ± 1.1 years. Dogs with G II tumors (*n* = 13) had tumors with an average size of 4.38 ± 0.97 cm, and an average age of 11.2 ± 0.65 years. Finally, dogs with G III tumors (*n* = 18) had tumors with an average size of 6.39 ± 1.36 cm, and an average age of 12.1 ± 0.54 years. These data clearly showed that canine mammary tumors develop more frequently in older dogs than in younger dogs, and that the malignancy of the tumor increased with the age of the dog.

### 3.2. Dogs with grade III tumors had a poor survival rate

**Figure 1:**
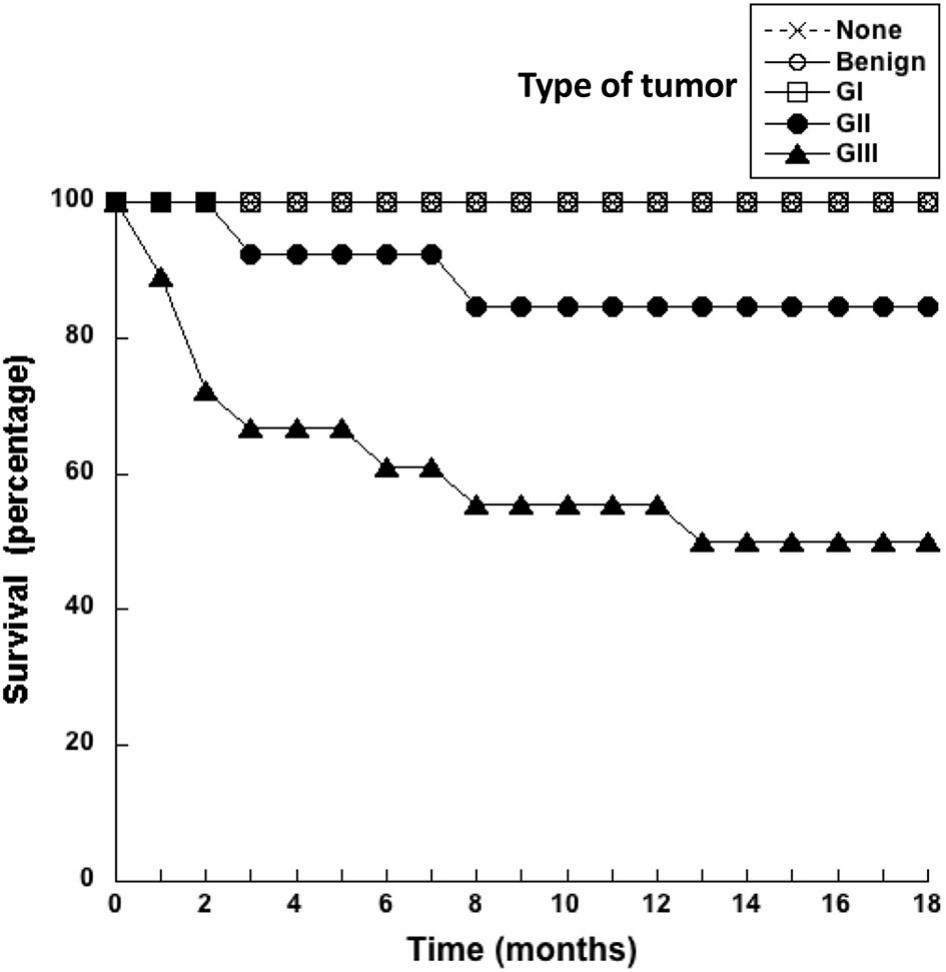
Dogs with grade III tumors had a poor survival rate. Healthy dogs (×) without tumors (none), and dogs with mammary tumors were monitored after surgery during 18 months. The survival of dogs with benign tumors, grade I (G I) tumors, grade II (G II) tumors, or grade III (G III) tumors was reported every month.

After surgery to remove the tumors, dogs were monitored every month for 18 months to assess whether they were still alive. Dogs did not receive any other treatment after surgery. The survival of dogs according to the type of tumor they had was registered monthly (Figure 1). All dogs with benign or G I tumors survived up to 18 months after surgery (Figure 1). In the group of dogs with G II tumors, one died 3 months after surgery, and another one died 8 months after surgery. Thus, by 18 months after surgery 84.6% of G II tumor-bearing dogs have survived (Figure 1). In contrast, only 50% of dogs with G III tumors were still alive at 18 months after surgery (Figure 1). These results showed again that animals with more aggressive tumors (G III) have a poor survival rate after surgery.

### 3.3. High neutrophil to lymphocyte ratio associates with more aggressive tumors

**Figure 2:**
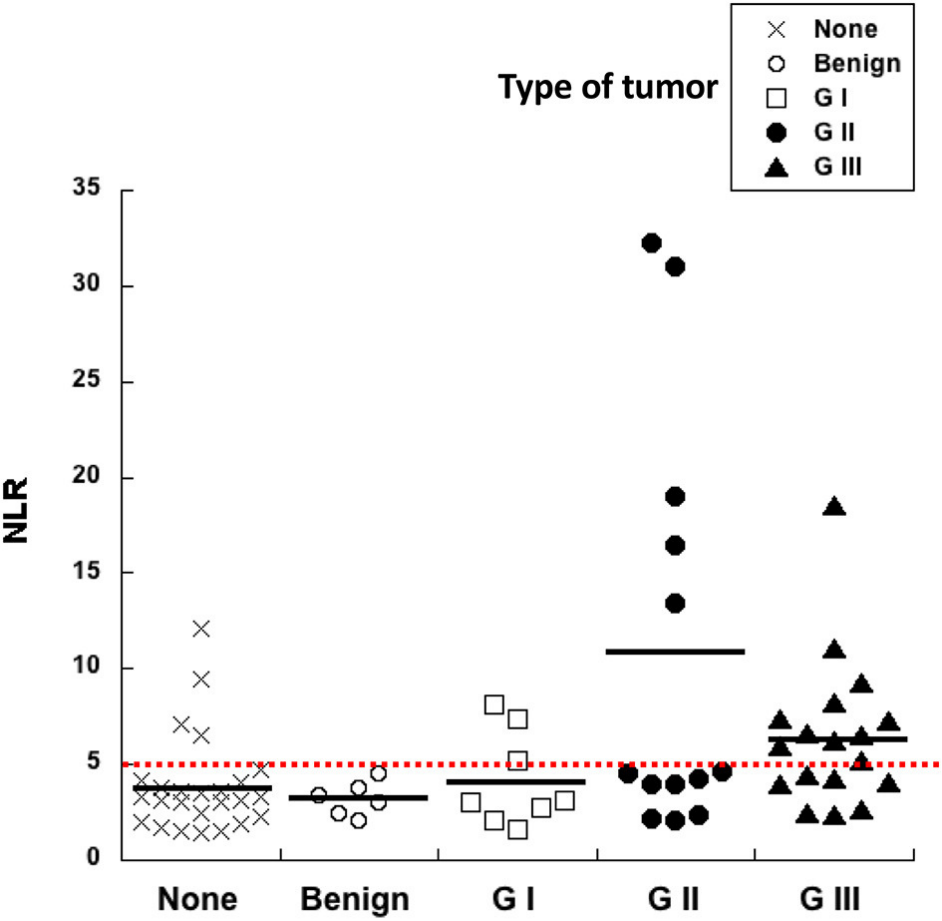
High neutrophil to lymphocyte ratio (NLR) correlates with more aggressive tumors. The neutrophil to lymphocyte ratio (NLR) calculated from blood analysis before surgery was compared to the histological grade of canine mammary tumors. Healthy dogs with no tumors (none), dogs with benign tumors, dogs with grade I (G I) tumors, dogs with grade II (G II) tumors, and dogs with grade III (G III) tumors. A cutoff value of NLR = 5 was calculated as a threshold separating healthy dogs from dogs probably bearing a tumor (red dotted line).

A pre-surgery indicator that would help predict whether an abnormal growth is more likely to be either benign or malignant would be very useful in veterinary medicine. Since inflammation is a condition that associates with cancer, cellular blood markers of systemic inflammation are good candidates as pre-surgery prognostic factors for cancer. The neutrophil to lymphocyte ratio (NLR) calculated from blood analysis before surgery was compared to the histological grade of all mammary tumors included in this study (Figure 2). The NLR of healthy control dogs was 3.8 ± 0.5 (Mean ± standard error, *n* = 25), while NLR of dogs with benign tumors was 3.2 ± 0.4 (Mean ± standard error, *n* = 6). Similarly, the NLR of dogs with G I tumors was 4.2 ± 0.9 (Mean ± standard error, *n* = 8). The aforementioned three groups showed no statistical difference among them (Figure 2). In contrast, the NLR of dogs with G II tumors was 10.3 ± 3.0 (Mean ± standard error, *n* = 13) (Figure 2), and the NLR of dogs with G III tumors was 6.4 ± 0.9 (Mean ± standard error, *n* = 18) (Figure 2). A contrast comparing normal, benign and G I groups vs. G II and G III groups showed that NLR values were statistically different. Clearly, higher pre-treatment NLR values can be used as a prognostic factor for tumors of higher grades.

**Figure 3:**
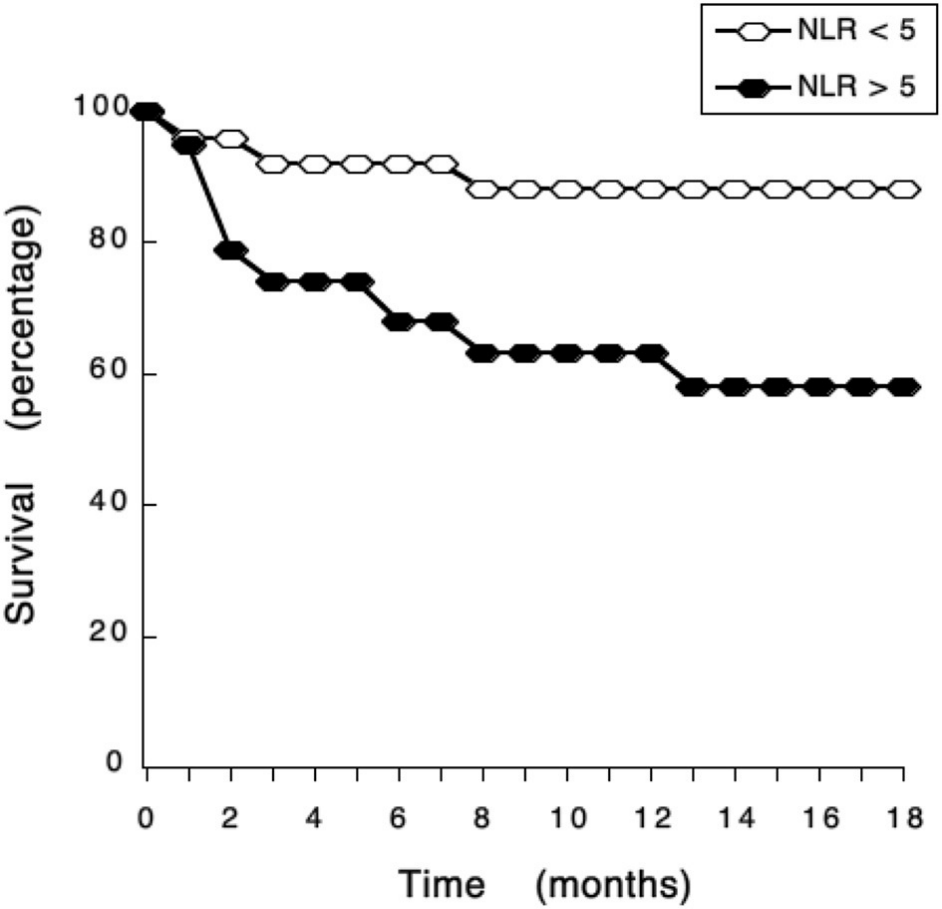
NLR exhibits a good prognostic value for the survival of dogs with mammary tumors. Dogs with mammary tumors and a neutrophil to lymphocyte ratio (NLR) <5, and dogs with mammary tumors and an NLR > 5 were monitored after surgery during 18 months. The survival rate was reported every month.

**Figure 4:**
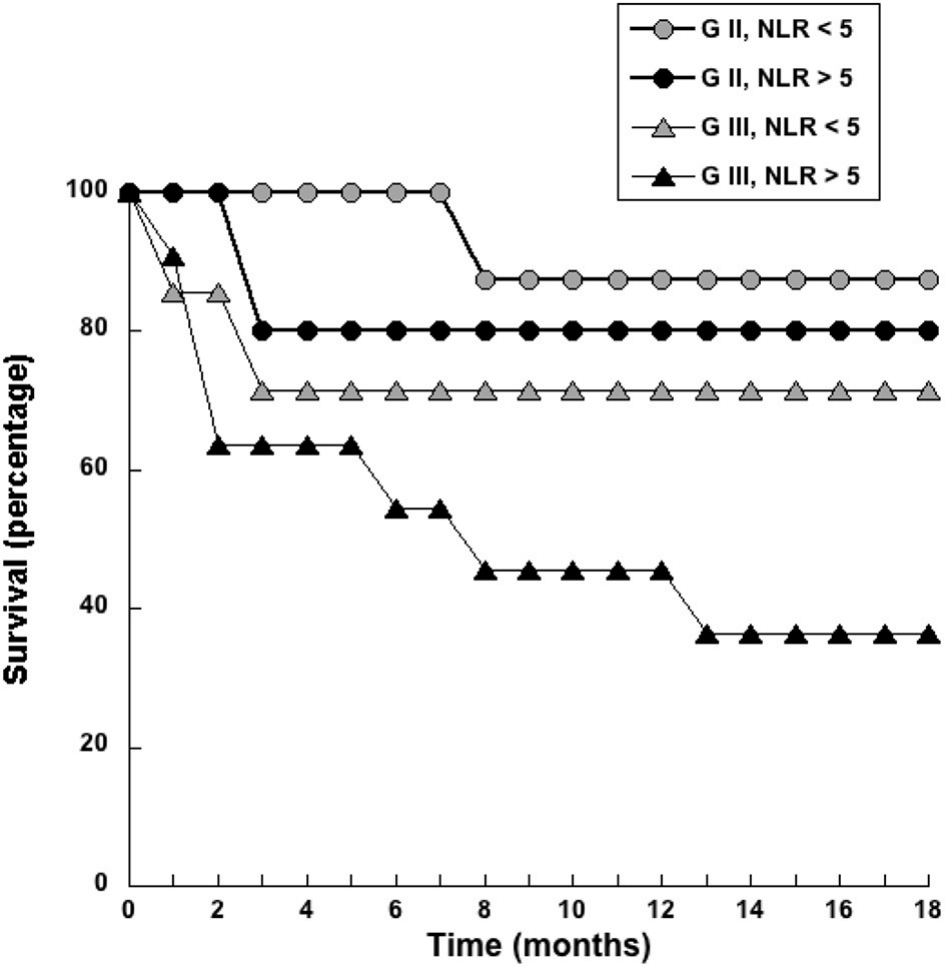
Dogs with a pre-treatment neutrophil to lymphocyte ratio (NLR) > 5 had a poor survival rate. Dogs with grade II (G II) or grade III (G III) tumors were separated according to their pre-treatment value in NLR <5 or NLR > 5. The survival rate was reported for each group every month during 18 months.

ROC curve analysis also identified a cutoff value of NLR = 5 (sensitivity 72.7%; specificity 74.6%) as a threshold separating surviving from not-surviving dogs (Figure 3). Nearly all dogs with NLR <5 had survived up to 18 months after surgery (Figure 3). In contrast, about 40% of dogs with NLR > 5 have not survived up to 18 months after surgery (Figure 3). The observed relative risk was 3.5 (95% CI 2.3–5.4), meaning that the risk of not surviving up to 18 months after surgery is 3.5 times greater for dogs with an NLR > 5 than for dogs with an NLR <5. Further analysis of dogs according to the grade of tumor they had, showed that among dogs with G II tumors and an NLR > 5, 20% have died by 3 months after surgery (Figure 4); while the majority of dogs with an NLR <5 had survived (Figure 4). For dogs with G III tumors and an NLR <5, about 30% have died by 3 months after surgery (Figure 4). In contrast, among dogs with GIII tumors and NLR > 5, 50% have already died by 6 months after surgery (Figure 4) and almost 70% have died after 1 year (Figure 4). Accordingly, these results showed that the pre-treatment NLR exhibits a good prognostic value for the survival of dogs with mammary tumors after surgery.

### 3.4. Albumin to globulin ratio did not correlate with tumor grade

**Figure 5:**
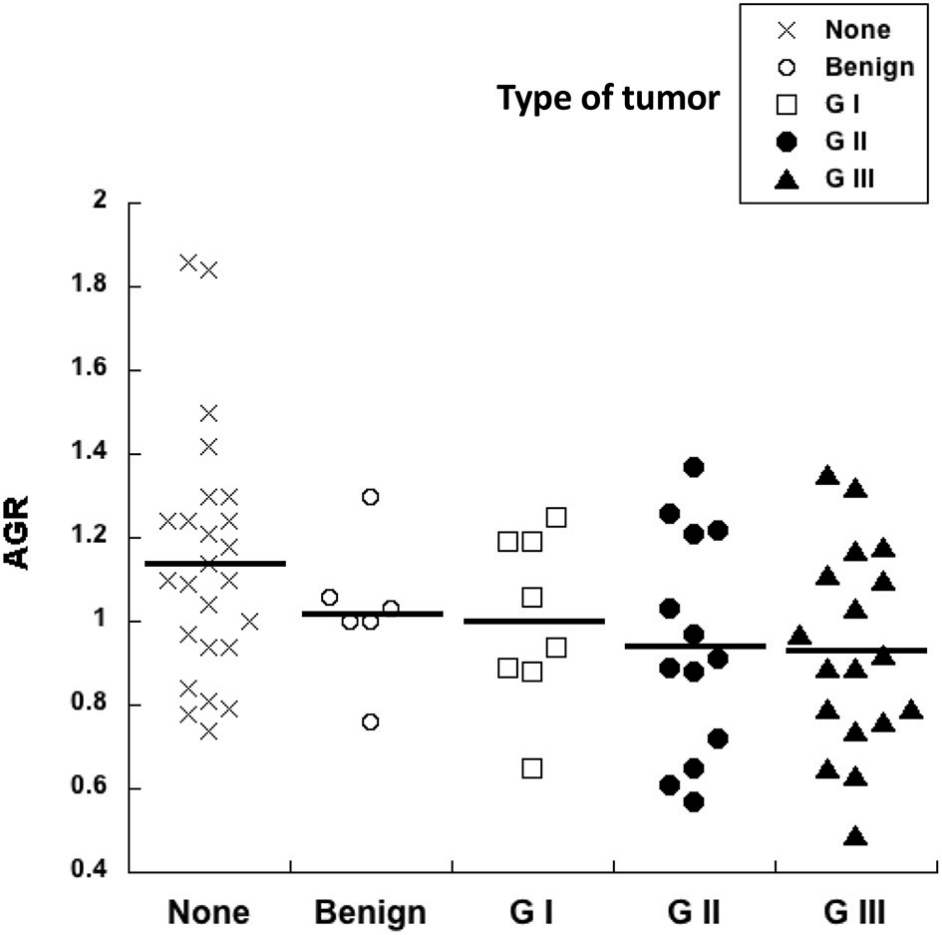
Albumin to globulin ratio does not correlate with tumor grade. The pre-surgery albumin to globulin ratio (AGR) calculated from blood analysis before surgery was compared to the histological grade of canine mammary tumors. Healthy dogs with no tumors (none), dogs with benign tumors, dogs with grade I (G I) tumors, dogs with grade II (G II) tumors, and dogs with grade III (G III) tumors. There was no statistical difference for AGR values among all these groups.

The pre-surgery AGR was calculated for every dog in our study. Healthy dogs had an AGR of 1.1 ± 0.05 (Mean ± standard error, *n* = 25) (Figure 5). All other dogs with tumors had similar AGR values (Figure 5). Although, a tendency to lower AGR values was observed in dogs with tumors, and even lower AGR values for dogs with G II and G III tumors, there was no statistical difference among all these groups (Figure 5). Therefore, the AGR alone does not provide any prognostic value for canine mammary tumors.

**Figure 6:**
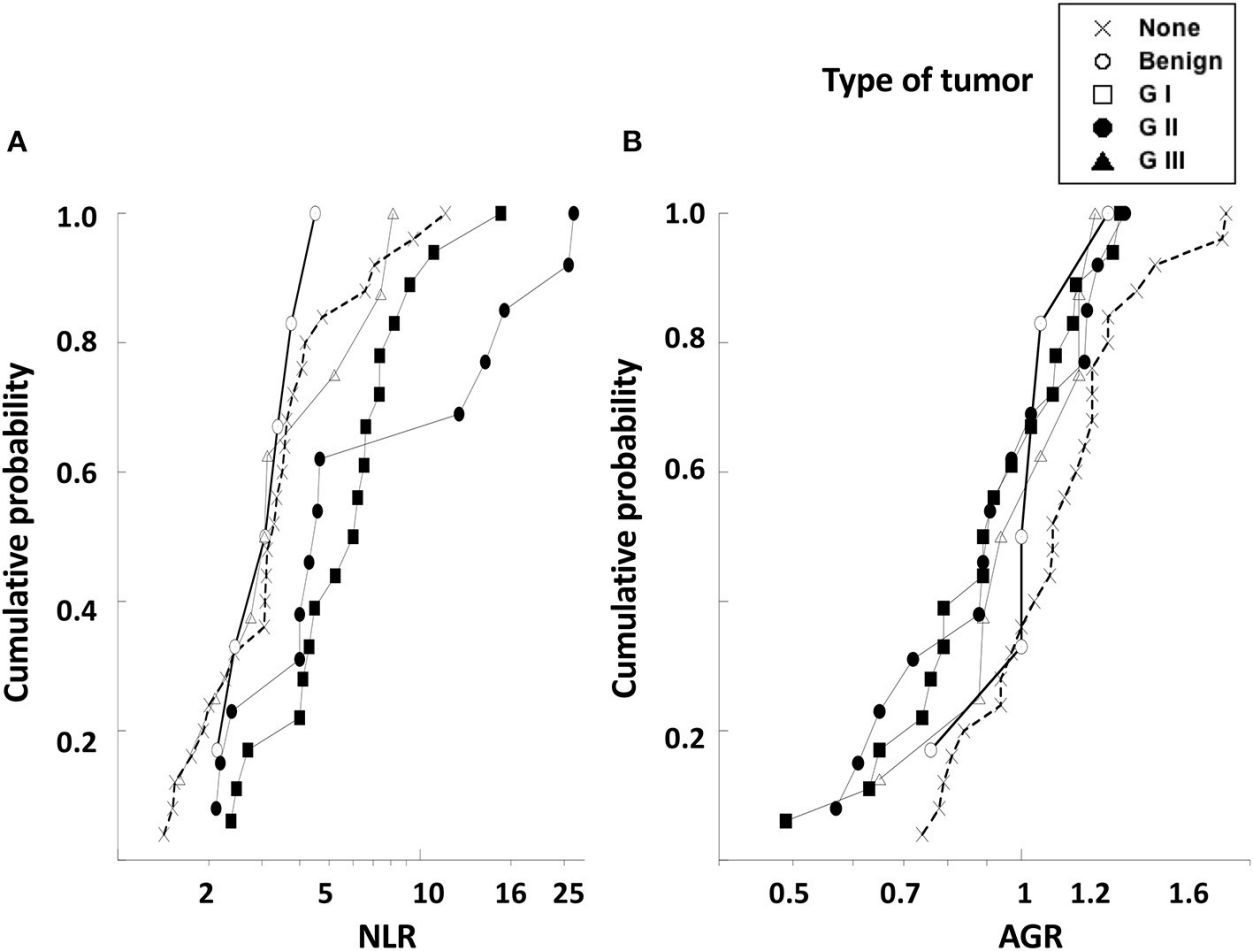
The empirical distribution function for NLR and AGR values of dogs with mammary tumors. The empirical distribution (cumulative probability) of pre-treatment values of **(A)** neutrophil to lymphocyte ratio (NLR) or of **(B)** albumin to globulin ratio (AGR) was calculated for healthy dogs with no tumors (none), dogs with benign tumors, dogs with grade I (G I) tumors, dogs with grade II (G II) tumors, and dogs with grade III (G III) tumors. The NLR values could distinguish between dogs with benign and G I tumors from dogs with G II and G III tumors. The AGR values were similar for all dogs independently of their health status. NLR and AGR are plotted on a log scale.

In order to better visualize differences among the variables NLR and AGR, the empirical distribution function, which is an estimate of the cumulative distribution (cumulative probability) of data in the sample (40, 41), was applied to our data. In this analysis, the value on the “*y*” axis at a given point is equal to the proportion of dogs in the sample whose NLR (Figure 6A) or AGR (Figure 6B) is less than or equal to the “*x*” value of that point. The NLR values could clearly distinguish two groups of dogs. NLR distributions of healthy dogs, dogs with benign and with GI tumors were very similar. In contrast, the NLR distributions of dogs with G II and G III tumors were shifted to the right, indicating that these dogs indeed have higher levels of NLR (Figure 6A). On the contrary, the AGR values were similar for all dogs independently of their health status (Figure 6B). These data indicated that the pre-treatment NLR was a good biomarker, although not sufficient, for predicting the type of tumor a dog had before surgery.

### 3.5. Principal component analysis offers prognostic advantage

**Figure 7:**
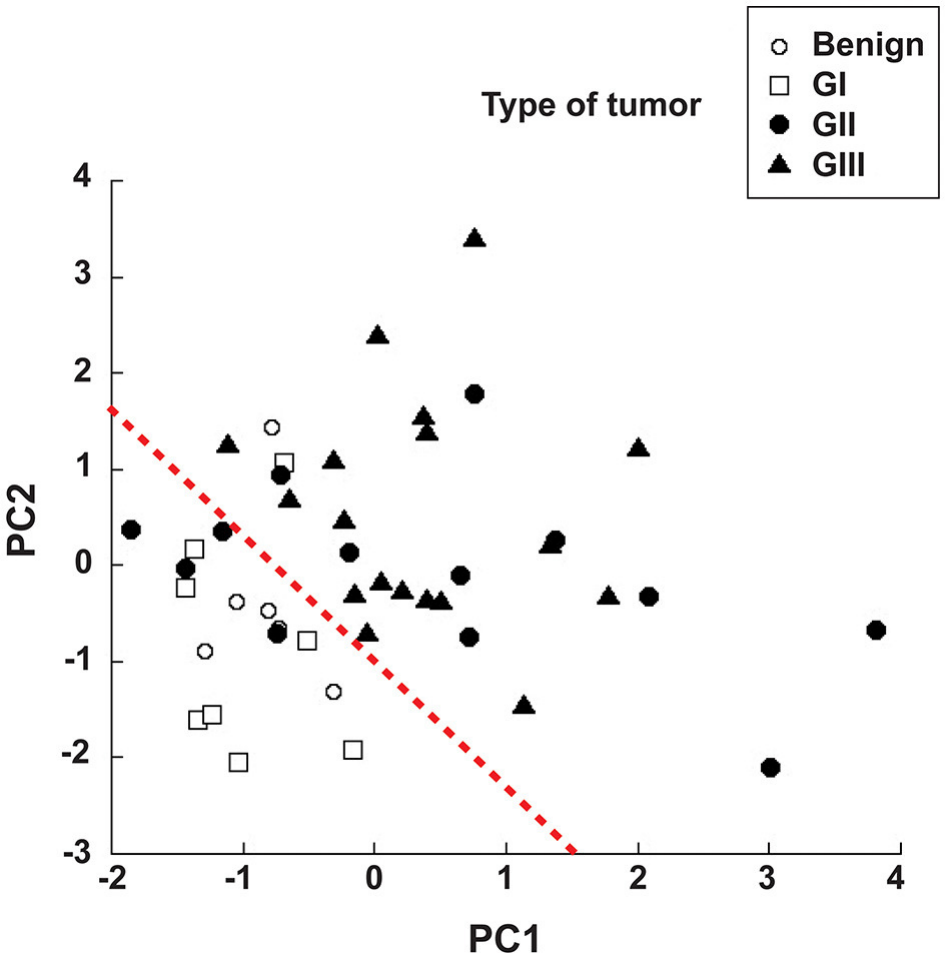
Principal component analysis offers prognostic advantage. The pre-treatment indicators neutrophil to lymphocyte ratio (NLR), albumin to globulin ratio (AGR), size of tumor, and age of dog were included in a principal component analysis (PCA). PCA identifies new variables, the principal components (PC1 and PC2), which are linear combinations of the original variables. The majority of dogs with G II tumors and all dogs with G III tumors clearly segregated apart (right side of the red dotted line) from dogs with benign and G I tumors (left side of red dotted line). The group of dogs at the left side of the dotted line was designated as having “good prognosis”, while the group at the right side of the dotted line was designated as having “poor prognosis”. The equations for the principal components are: PC1 = (0.397) (age in years) + (0.0658) (NLR) – (0.588) (AGR) + (0.252) (tumor size in cm), and PC2 = (0.563) (age in years) – (0.226) (NLR) + (0.418) (AGR) + (0.676) (tumor size in cm).

**Figure 8:**
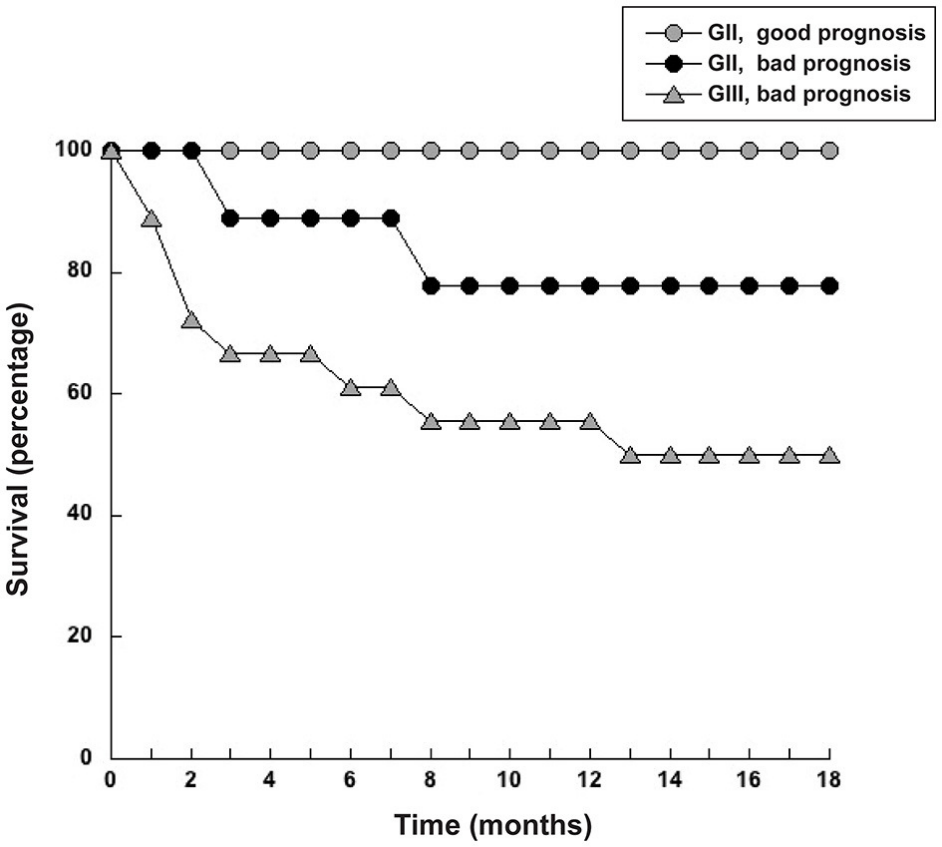
Dogs with grade II tumors and within the good prognosis group had a higher survival rate. Dogs with grade II (G II) tumors that segregated either within the good prognosis group or the bad prognosis group defined in the principal components analysis (PCA) were monitored for survival after surgery for 18 months. Dogs with grade III (G III) tumors in the poor prognosis group had the lowest survival rate.

**Figure 9:**
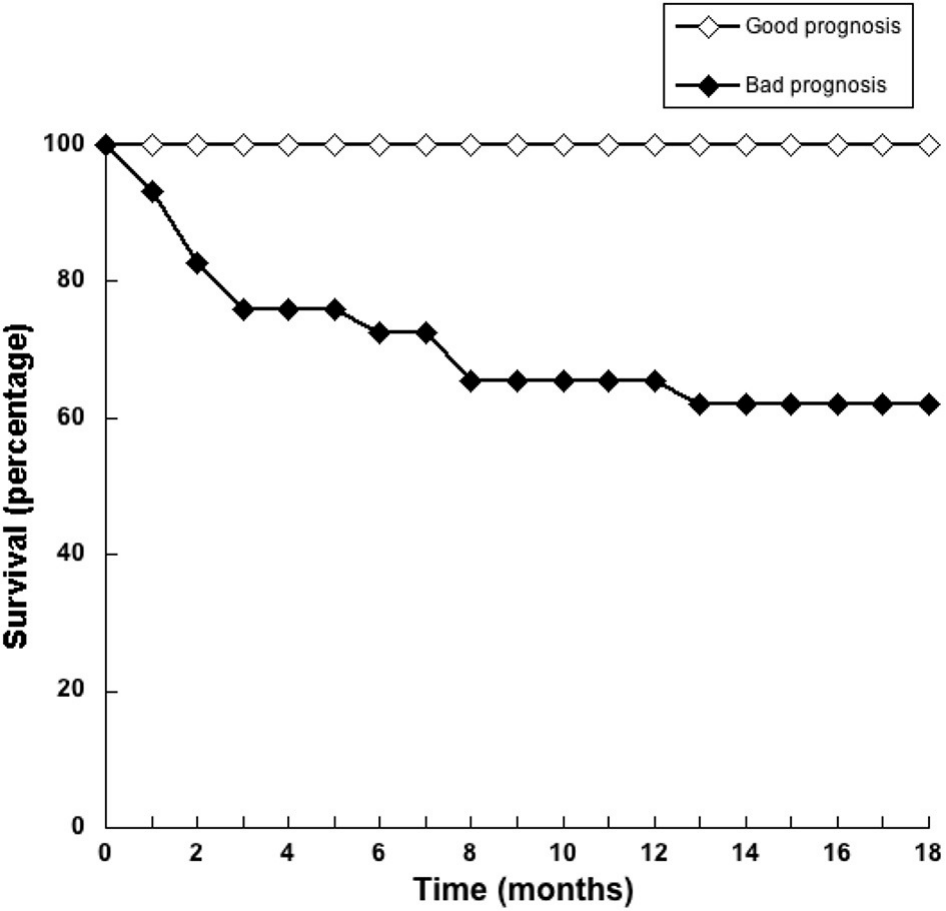
Pre-treatment principal component analysis provide a useful predictive tool for dogs with mammary tumors. Principal component analysis (PCA) of simple pre-treatment indicators segregates dogs in two groups, namely good prognostic or bad prognostic groups. The survival rate of dogs in each group was estimated for 18 months after surgery. All dogs in the good prognosis group (white symbols) had a high survival rate. Dogs in the poor prognosis group (black symbols) had a much lower probability of survival.

Since NLR was not sufficient for predicting the type of tumor, we explored whether combining several pre-treatment indicators could offer a better prognostic tool. For this, the indicators NLR. AGR, size of tumor, and age of dog were included in a principal component analysis (PCA). PCA identifies new variables, the principal components, which are linear combinations of the original normalized variables. The principal components with Eigen values higher than one were defined as: PC1 = (0.397) (age in years) + (0.0658) (NLR) – (0.588) (AGR) + (0.252) (tumor size in cm), and PC2 = (0.563) (age in years) – (0.226) (NLR) + (0.418) (AGR) + (0.676) (tumor size in cm). These first two principal components explained 72.2% of total data variability. As a result, much of the variation existing in the data was reduced to two dimensions (Figure 7). This analysis showed that the majority of dogs with G II tumors and all dogs with G III tumors clearly segregated apart from dogs with benign and G I tumors (Figure 7). The group of dogs at the left side of the dotted line was designated as having “good prognosis”, while the group at the right side of the dotted line was designated as having “poor prognosis” (Figure 7). All dogs in the “good prognosis” group survived up to 18 months after surgery. The observed relative risk was 3.8 (95% CI 2.3–6.3), meaning that the risk of not surviving up to 18 months after surgery is 3.8 times greater for dogs in the “poor prognosis” group than for dogs in the “good prognosis” group. Interestingly, the one dog with a benign tumor and the one dog with a G I tumor that segregated in the poor prognosis group were the oldest dogs (13 and 12 years old, respectively) and had the largest tumors in their group (Table 1). Among the dogs with G II tumors, four segregated within the good prognosis group. All these dogs survived up to 18 months after surgery (Figure 8). In contrast, the dogs with GII tumors that segregated within the poor prognosis group presented a lower survival rate (Figure 8). Similarly, all dogs with G III tumors were in the poor prognosis group and had the lowest survival rate (Figure 8). Hence, with only simple pre-treatment indicators, the PCA is capable of predicting a good survival rate for the dogs segregating in the good prognosis group, independently of the grade of tumor they have (Figure 9). Conversely, a dog segregating in the poor prognosis group has a much lower probability of survival (Figure 9). Together, these results confirm that pre-treatment PCA provide a useful predictive tool for canine mammary tumors.

## 4. Discussion

Inflammation is now recognized as a hallmark for cancer development (17, 18) because it influences each step of tumorigenesis, including tumor initiation, growth, and metastatic progression (19, 20). In consequence, cellular and biochemical blood markers of systemic inflammation, such as the neutrophil to lymphocyte ratio (NLR) and the albumin to globulin ratio (AGR) have been proposed as prognostic factors for human cancer development (21, 22). These biomarkers have not been explored as prognostic factors for cancer development in veterinary medicine. Therefore, in this study we investigated the prognostic value of NLR and AGR in canine mammary tumors. In addition, other clinical data such as age, breed, tumor size, and survival time after surgery were considered. It was found that a higher pre-treatment NLR value (NLR > 5) was associated with a less survival rate. In contrast, the AGR did not show any predictive value on the malignancy of tumor. Multivariate logistic regression could then be used to analyze our data. Unfortunately, in order to perform this analysis with four explanatory variables, a much larger sample size than the one we had, was required. For this reason, a principal component analysis (PCA) (39) was performed instead. By combining the NLR with other parameters, such as AGR, age of the dog, and tumor size, in a PCA, the grade of the tumor and survival after surgery could be appropriately predicted. Thus, pre-treatment NLR values have a prognostic value for the survival rate after surgery of dogs with mammary tumors. Also, the NLR in combination with other indicators in a principal component analysis can be even a better predictor of the grade of the tumor and survival after surgery.

Dogs are nowadays more than just companion animals, and are considered family members. As such, their health is an important issue for their owners. Dogs, similarly to humans, develop spontaneously different types of cancer (2, 4) exhibiting a global incidence of around 50% (7–10). Cancer is the leading cause of death among family dogs, with mammary gland tumors being the most common type of cancer (1). Just as with humans, an early diagnosis allows for better therapeutic options. However, for canine mammary tumors, the best diagnosis method continues to be the pathological examination of the suspected tissue (12). This entails a biopsy or surgical removal of the tissue (13), imposing not only clinical and technical difficulties but also economic burden. Therefore, there is a lot of interest in finding ways that could predict the biological behavior of a tumor, before performing any surgery. A biomarker suggesting whether a tumor is more likely to be either benign or malignant would be a very useful tool deciding what the best therapeutic options are (14). Several clinical parameters have been considered as potential indicators of the biological behavior of a tumor, including age, race of the dog, and tumor size (42). Unfortunately, these parameters are susceptible to varying degrees of subjectivity and consequently none of them has proven to be useful (11, 15).

Biochemical and cellular indicators of systemic inflammation are good candidates as pre-surgery prognostic markers for cancer. For example, elevated C-reactive protein (CRP) (43), globulin (22), and certain cytokines (44) have been used as prognostic tools in patients with cancer. These substances however are not easy to measure since they require special equipment and expensive reagents. Instead, leukocytes can be more easily and conveniently measured than biochemical markers (45). In systemic inflammation, lymphopenia and neutrophilia are commonly seen (46). Lymphocytes play a relevant role in immune surveillance of tumors, and particularly cytotoxic T lymphocytes are important anti-tumor effector cells (47). Neutrophils are the first leukocytes to respond to inflammatory conditions (48, 49) and may promote tumor growth in several ways (50–52). Based on these findings and the fact that peripheral blood leukocytes are easily available, neutrophil and lymphocyte counts have been explored as markers of inflammation and immune response in cancer patients. However, independent leukocyte counts cannot provide a clear picture of the whole inflammatory state of an individual. A better picture is obtained when variations in several leukocytes are considered together. To this end, a particular parameter, the neutrophil to lymphocyte ratio (NLR) seems much more useful in predicting the inflammatory state.

The NLR can indicate an inflammatory condition by relating both changes in neutrophils and lymphocytes at the same time, and hence it is more useful than either neutrophil count or lymphocyte count alone, as the ratio reduces the effect of variations in either cell type (53). An elevated NLR is thought to exhibit the protumor activity of neutrophils and the reduced antitumor activity of lymphocytes (26). In addition, because NLR can be easily calculated from peripheral blood test results, it is also relatively inexpensive. Consequently, NLR is a promising marker to predict the outcome of cancer. Many studies have shown the correlation between high pretreatment NLR values and poor prognosis in many types of cancer (45, 54, 55). Particularly, in early breast cancer, higher NLR values associate with worse outcome in multiple ethnic groups (56). However, despite the fact that NLR has proven to be a good pre-surgery biomarker with prognostic value in human breast cancer, in the case of companion animals with canine breast mammary tumors, the NLR has not been explored. Few studies have shown a correlation between higher NLR values and some types of cancer in dogs, including sarcoma (26), mast cell tumors (13), lymphomas (24), and oropharyngeal tumors (57).

Now, we report that also in mammary tumors NLR is indeed a good prognostic biomarker for the outcome of canine mammary tumors after surgery. In healthy dogs the NLR was 3.8. However, NRL values increased in dogs with higher grade tumors. These dogs also showed a poor survival rate after surgery. A threshold value of NLR = 5 was established to separate dogs with no tumors and with low-grade tumors from dogs with more aggressive (grade II and grade III) tumors (Figure 2). With this value, it was found that the risk of not surviving up to 18 months after surgery in dogs with an NLR > 5 is 3.5 times higher than the risk of dogs with an NLR <5 (Figure 3). This indicated that pre-treatment NLR alone can predict the outcome of many dogs. Therefore, NLR becomes an easy and inexpensive tool that could help making therapeutic decisions right from the start. In the case of dogs, this becomes a valuable indicator because a proper diagnosis always requires a more invasive and usually expensive procedure (biopsy and or surgery). However, NLR alone is not an ideal biomarker (42). About half of the dogs with NLR > 5 had a good survival rate after surgery. There is no easy way to distinguish these animals from the ones that had a poor outcome. This is not surprising since NLR being primarily an inflammation biomarker is influenced by many other factors (e.g., infection, strenuous exercise, stress, etc.) (58). In fact, inflammation in any type of cancer is extremely complex with both stimulation and inhibition elements playing a role at the same time in the tumor microenvironment (59). Still, NLR remains a good indicator of poor prognosis that will help both the veterinarian and dog owner to make better decisions.

In addition to NLR, cytological examination of suspected tissue remains as a routine simple, and relatively inexpensive method utilized as pre-surgical diagnosis of canine mammary tumors (60, 61). Cytological examination is important for establishing the histopathological grade of malignancy and by this means estimate the prognosis (12). However, due to the heterogeneous morphology of canine tumors, cytological examination exhibits relatively lower sensitivity and specificity than examination of human tumors (60). Still, when proper samples are obtained, the accuracy of diagnosing malignancy can be as high as 95% (61). Nevertheless, when samples of tissue are obtained by fine-needle aspiration, cytological diagnosis of canine mammary tumors suffers from relatively low accuracy and sensitivity (around 60%) because needle samples usually do not provide a complete vision of the tissue, particularly for complex or mix tumors (61). Therefore, even though cytological examination remains as the gold standard for diagnosis of canine mammary tumors (12), it would be helpful to have other means to foresee the behavior of the tumor, before performing any surgery. This is particularly important in cases of older dogs and when dog owners' finances are limited.

Other parameters have also been explored as possible indicators of severity of disease in canine cancers. Among these parameters, tumor size, age of the dog, and some biochemical indicators have been considered (16, 22, 42). Our own data showed that canine mammary tumors develop more frequently in older dogs than in younger dogs, and that the malignancy of the tumor increased with the age of the dog. Thus, tumor size and age of the dog are clearly connected to the malignancy of the tumor. Also, the albumin to globulin ratio (AGR) has been proposed to have a predictive value for cancer patients (22, 62). In veterinary medicine, AGR was altered during bacterial infections in cats (63) and during parasitic infections in dogs (64). But there are not reports on AGR in canine mammary tumors. Now, we report here that the AGR did not change among dogs with or without mammary tumors. Therefore, the AGR alone does not provide any prognostic value for canine mammary tumors. Nonetheless, combining NLR and AGR was proposed to have a better predictive value in patients with triple negative breast cancer (65). Consequently, we explored whether combining several pre-treatment indicators could offer a better prognostic tool.

The principal component analysis (PCA) is a method that transforms a large set of variables into a smaller one that still contains most of the information in the large set (38). PCA allows for the identification of new variables, the principal components, which are linear combinations of the original normalized variables (39). We used PCA to combine the easily accessible indicators NLR, AGR, size of tumor, and age of dog. As a result, two principal components, PC1 and PC2, were defined, reducing in the process our data to two dimensions (Figure 7). This analysis showed that the majority of dogs with G II tumors and all dogs with G III tumors clearly segregated apart from dogs with benign and G I tumors (Figure 7). Thus, we defined two groups of dogs, one with “good prognosis” and another with “poor prognosis” (Figure 7). All dogs in the “good prognosis” group presented a good survival rate, independently of the grade of tumor they had (Figure 9). In contrast, dogs in the “poor prognosis” group has a much lower probability of survival (Figure 9). As a result, the combination of a few simple pre-treatment indicators in the PCA is capable of predicting a good survival rate for dogs with canine mammary tumors. We propose that the use of the principal components defined in this study could help veterinarians and dog owners to make better pre-treatment decisions about the health of the dog.

There are some limitations in this study. First, this is a retrospective analysis from a single institution, the number of cases included is relatively small, and the time of recruitment was also short (18 months). Second, patients varied considerably in age, breed, and disease stage, and consequently also in immune responses. Due to the small number of patients, we could not conduct an analysis of these dog subgroups. A large number of dogs were excluded from further analysis due to incomplete data, or diagnosis of a different type of tumor. Third, analysis of the relationship between NLR and overall survival after surgery was limited to the information in clinical records. We did not have access to patients after surgery. Furthermore, this study did not evaluate actual tumor-associated neutrophils or lymphocytes in parallel to peripheral blood counts to determine NLR. Future studies, should include analysis of both circulating leukocytes and tumor-associated neutrophils. The limitations of this study mean that prospective studies should be conducted to further validate the NLR threshold value recommended, as well as the predictive value of the PCA.

In conclusion, we have found that a higher pre-treatment NLR value (NLR > 5) is associated with less survival rate of dogs with mammary tumors, suggesting that NLR can be used as a prognostic marker for disease severity. In addition, by combining the NLR with AGR, age of the dog, and tumor size in a principal component analysis (PCA), a much stronger predictive tool was developed. With the PCA the grade of the tumor and survival after surgery could be more accurately predicted. These simple indicators should be used in the clinic to improve the decision making regarding possible treatments.

## Data availability statement

The original contributions presented in the study are included in the article/supplementary material, further inquiries can be directed to the corresponding author.

## Ethics statement

Approval by the Committee for the Care of Experimental Animals was not required, since all dogs underwent laboratory tests and surgery for medical treatment with the approval from their owners. Written informed consent for participation was not obtained from the owners because the data in this study was obtained from clinical records.

## Author contributions

EU-Q designed the research, analyzed data, and prepared figures. LR-R provided all clinical records and performed the histopathological analysis. TG performed graphical and statistical analysis. CR designed the research, analyzed data, organized the references, and wrote the paper. All authors reviewed and approved the manuscript.
