# Corrigendum: Neutrophil to lymphocyte ratio and principal component analysis offer prognostic advantage for dogs with mammary tumors

**DOI:** 10.3389/fvets.2024.1395930

**Published:** 2024-05-02

**Authors:** Eileen Uribe-Querol, Laura Romero-Romero, Tzipe Govezensky, Carlos Rosales

**Affiliations:** ^1^Laboratorio de Biología del Desarrollo, División de Estudios de Posgrado e Investigación, Facultad de Odontología, Universidad Nacional Autónoma de México, Mexico City, Mexico; ^2^Departamento de Patología, Facultad de Medicina Veterinaria y Zootecnía, Universidad Nacional Autónoma de México, Mexico City, Mexico; ^3^Apoyo de estadística, Instituto de Investigaciones Biomédicas, Universidad Nacional Autónoma de México, Mexico City, Mexico; ^4^Departamento de Inmunología, Instituto de Investigaciones Biomédicas, Universidad Nacional Autónoma de México, Mexico City, Mexico

**Keywords:** neutrophil, lymphocyte, breast cancer, canine mammary tumor, inflammation, cancer, albumin, globulin

In the published article, there was an error. In section 3.3, data on separation of low-grade from high-grade tumors was referred instead of the correct data on separation of surviving from not- surviving dogs.

A correction has been made to Section 3.3 High neutrophil to lymphocyte ratio associates with more aggressive tumors; Second paragraph. The sentence previously stated: “ROC curve analysis identified a cutoff value of NLR = 5 (sensitivity 72.7%; specificity 74.6%) as a threshold separating low-grade from high-grade tumors (Figure 2).”

The corrected sentence appears below:

“ROC curve analysis also identified a cutoff value of NLR = 5 (sensitivity 72.7%; specificity 74.6%) as a threshold separating surviving from not-surviving dogs (Figure 3).”

The authors apologize for this error and state that this does not change the scientific conclusions of the article in any way. The original article has been updated.

